# Pyoderma Gangrenosum After Bilateral Total Knee Arthroplasty

**DOI:** 10.1016/j.artd.2021.07.003

**Published:** 2021-08-26

**Authors:** Reilly Loomis, Mellanie Merrit, Maria Alexandrovna Aleshin, Grace Graw, Gordon Lee, Bradley Graw

**Affiliations:** a Palo Alto Medical Foundation, Center for Total Joint Replacement, Palo Alto, CA; b Sequoia Hospital, Center for Joint Replacement, Redwood City, CA; c Department of Dermatology, Stanford University Medical Center, Palo Alto, CA; d Department of Plastic and Reconstructive Surgery, Stanford University Medical Center, Palo Alto, CA

**Keywords:** Total knee arthroplasty, Pyoderma gangrenosum, Prosthetic joint infection, Necrotizing fasciitis, Pathergy

## Abstract

Pyoderma gangrenosum is a neutrophilic dermatosis, which mimics both infection and necrotizing fasciitis, that can present after surgical interventions. We present the case of a 62-year-old male who underwent one-stage bilateral total knee arthroplasty. Nine days after the surgery, he presented with wound breakdown, high fever, and elevated white blood cell count. Repeated debridement was performed, and empiric antibiotics were given. All tissue cultures and aspirates remained negative throughout treatment course, and the patient remained unresponsive to therapy. The patient was eventually diagnosed with pyoderma gangrenosum after infectious etiologies were ruled out and after a skin biopsy and dermatologic consultation. His condition rapidly improved after treatment with corticosteroids, and soft-tissue defects were repaired with skin substitute and full-thickness skin grafting. In patients with aseptic wound breakdown after total knee arthroplasty, pyoderma gangrenosum is a rare but devastating complication and should be considered.

## Introduction

Pyoderma gangrenosum (PG) is a dermatosis characterized by the elevated presence of neutrophils in the absence of infection [[1], [2], [3]]. It can be idiopathic or present secondary to trauma or surgical intervention. Both the idiopathic and secondary forms of PG are exceedingly rare with a worldwide incidence of 3 to 10 patients per million population [4,5]. An underlying inflammatory or neoplastic disease is present in 50%-78% of these cases [5,6,7]. A PG lesion typically begins as a nodule or pustule that rapidly progresses into an ulceration with violaceous borders and marginal erythema [8]. Overall, these lesions are most common in the lower extremities but postoperatively are more likely to involve the breast or abdomen [7,9]. Owing to its clinical appearance and correlation to surgical intervention, it is commonly misdiagnosed as postoperative wound infection or necrotizing fasciitis, leading to 73% patients being treated with wound debridement and 90% with empiric antibiotic therapy [9,10]. In this case report, we discuss a case of a man with postoperative PG after bilateral total knee arthroplasty.

## Case history

**Figure 1:**
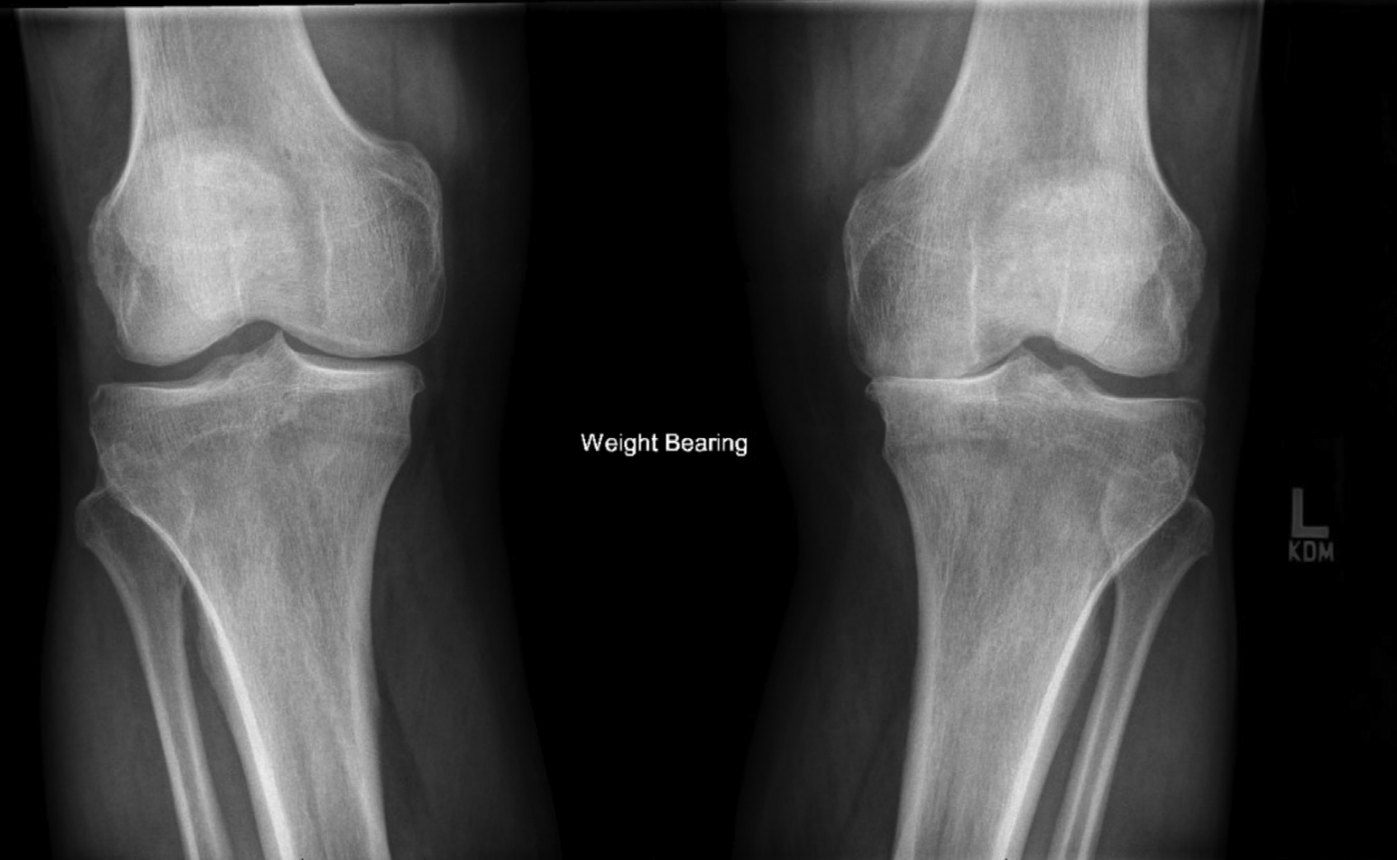
Bilateral standing anterior-posterior weight bearing knee radiographs.

**Figure 2:**
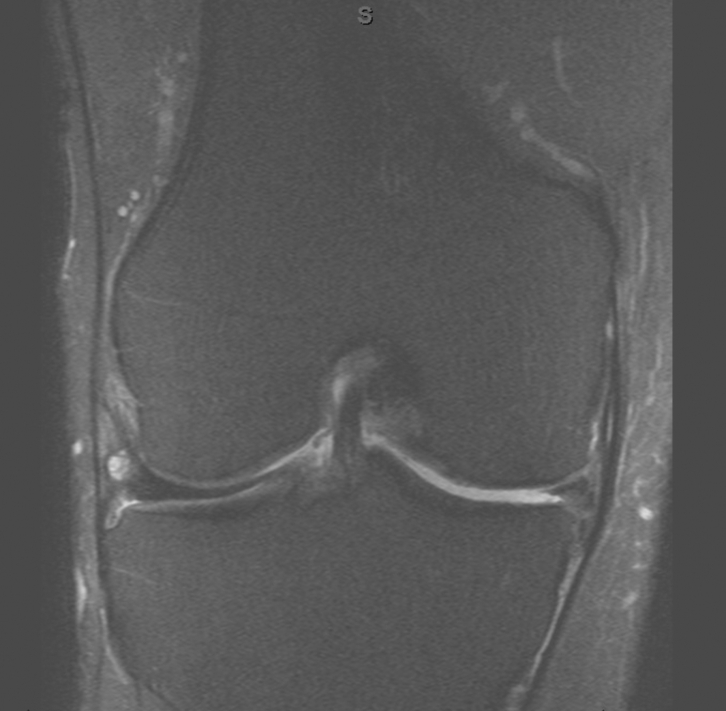
Magnetic resonance imaging of the left knee showing medial compartment chondromalacia.

A 62-year-old male with a past medical history of hypertension, hyperlipidemia, and paroxysmal atrial fibrillation presented to our orthopedic center for joint replacement with long-standing bilateral knee osteoarthritis. In addition, he has class 1 obesity, with a body mass index of 32 (kg/m^2^), and has worked as a high-technology machinist in Silicon Valley noting years of discomfort limiting his walking ability. With severe medial compartment and patellofemoral osteoarthritis, he was interested in proceeding with bilateral total knee replacement (Figure 1, Figure 2). The decision for bilateral total knee replacement was based on patient request given symmetric deformity and pain and clearance from his cardiologist. Informed consent, including a conversation about increased risk of bilateral compared with unilateral total knee replacement, was obtained for the procedure as well as for the writing of this report [11]. The patient underwent bilateral total knee arthroplasty through standard medial parapatellar arthrotomy using Zimmer NexGen cruciate-retaining knee replacement prostheses (Warsaw, IN) without intraoperative complication. Anesthetic technique included spinal, intravenous sedation, and adductor canal block placed under ultrasound guidance. A cemented technique was used with patellar resurfacing, prophylactic cefazolin, IV tranexamic acid, sequential tourniquet inflation, and total operative time for bilateral knee replacements of approximately 3 hours. Arthrotomy closure was performed with 1-PDS Ethicon (Somerville, NJ) suture, the dermis with 2-0 PDS suture, and the skin with 3-0 Prolene suture. Initial bandages included Steri-Strips, nonadherent gauze, sterile 4 × 8 gauze sponge, and compression wrap.

**Figure 3:**
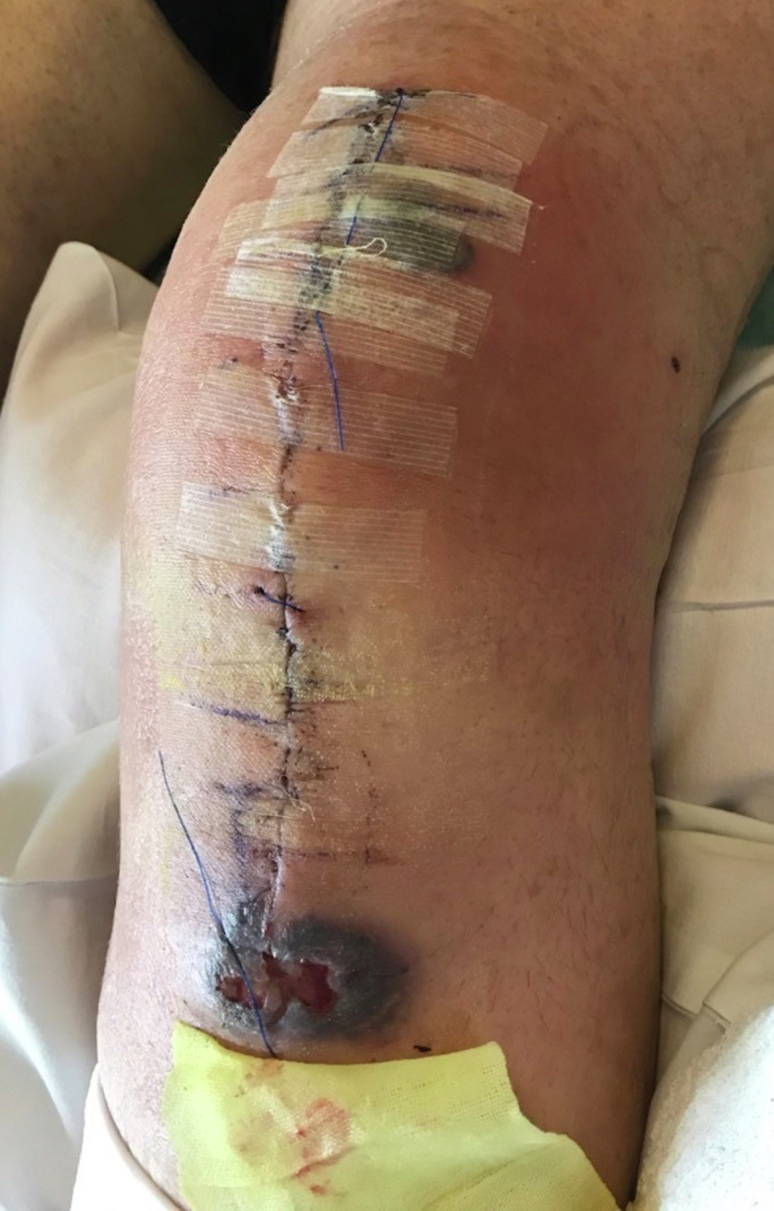
Left knee presentation on postoperative day #13, notice violaceous inferior wound border.

**Figure 4:**
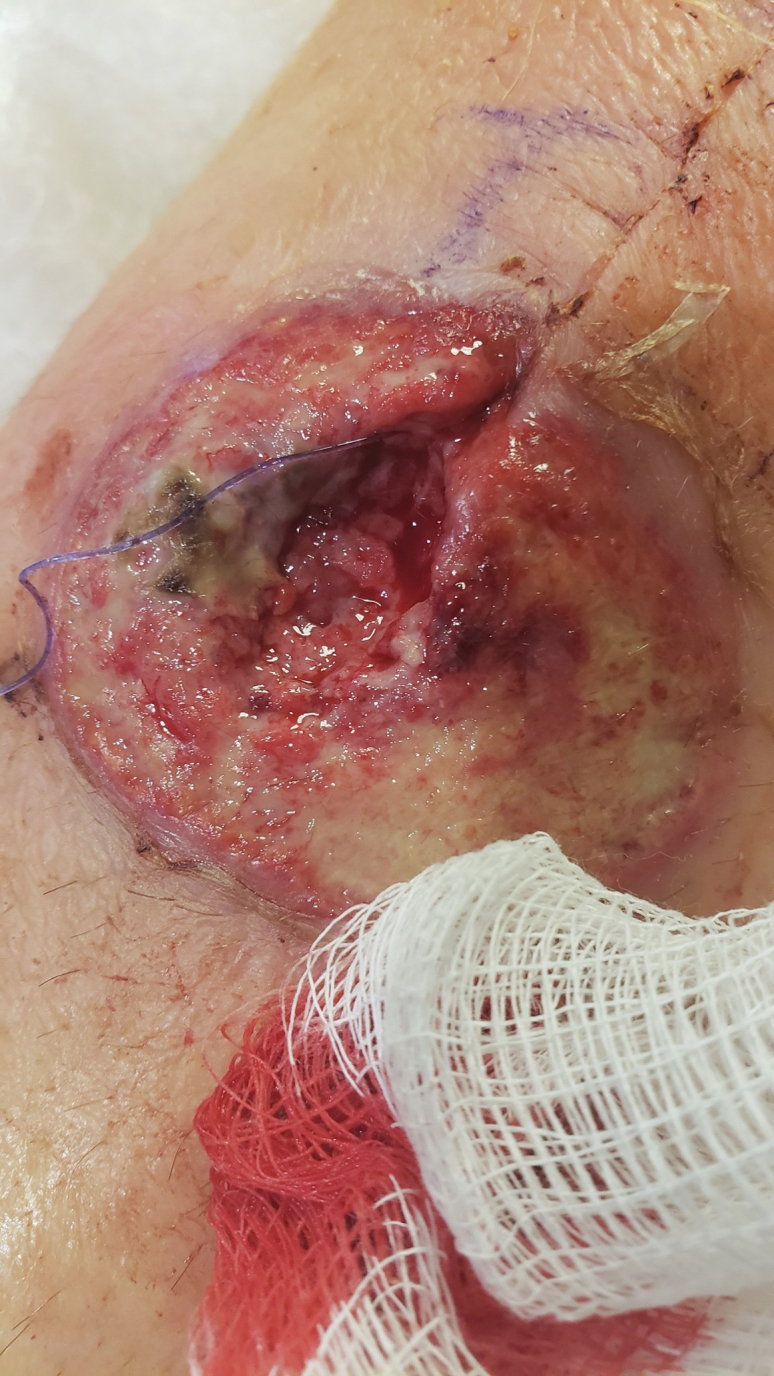
Left knee inferior wound after local debridement.

On postoperative day two, the patient aspirated secondary to narcotic-induced delirium. A chest computed tomography showed pneumonitis and subsegmental pulmonary embolism. His symptoms resolved with supportive respiratory care, and IV enoxaparin was started for treatment of the pulmonary embolism. He was subsequently discharged on postoperative day six with oral antibiotics and anticoagulant until further notice. One week later, he presented to a nearby emergency department with high fevers (exceeding 101.5°F) and left knee redness with a white blood cell (WBC) count of 18. He was not given any antibiotics and was told to see his surgeon the next morning. He presented to clinic with continued fevers and left knee superficial dehiscence at its distal aspect (Fig. 3). His right knee had no signs of infection, hematoma, or wound breakdown at this time. Given the progressive nature of his symptoms, he was admitted to the hospital and placed on IV antibiotics (vancomycin and piperacillin/tazobactam) with twice daily dressing changes. His inflammatory markers were elevated on postoperative day 10 with an erythrocyte sedimentation rate of 106 and C-reactive protein of 21.6. The knee was not aspirated at that time given a low suspicion of deep infection. On postoperative day 15, his condition stabilized including normalization of his WBC, and an inferior vena cava filter was placed to limit anticoagulant dosage. However, on postoperative day 18, his condition deteriorated with recurrence of fevers >101.5°F and increasing WBCs to 13.3 despite continued IV antibiotic therapy. His knee wounds progressively worsened, with areas of soft-tissue necrosis now involving the right knee (Fig. 4). Aspirate was obtained from both knees: right—5000 WBC, 44% polymorphonuclear leukocytes (PMNs), gram stain negative; left—1500 WBC, 78% PMNs, gram stain negative.

**Figure 5:**
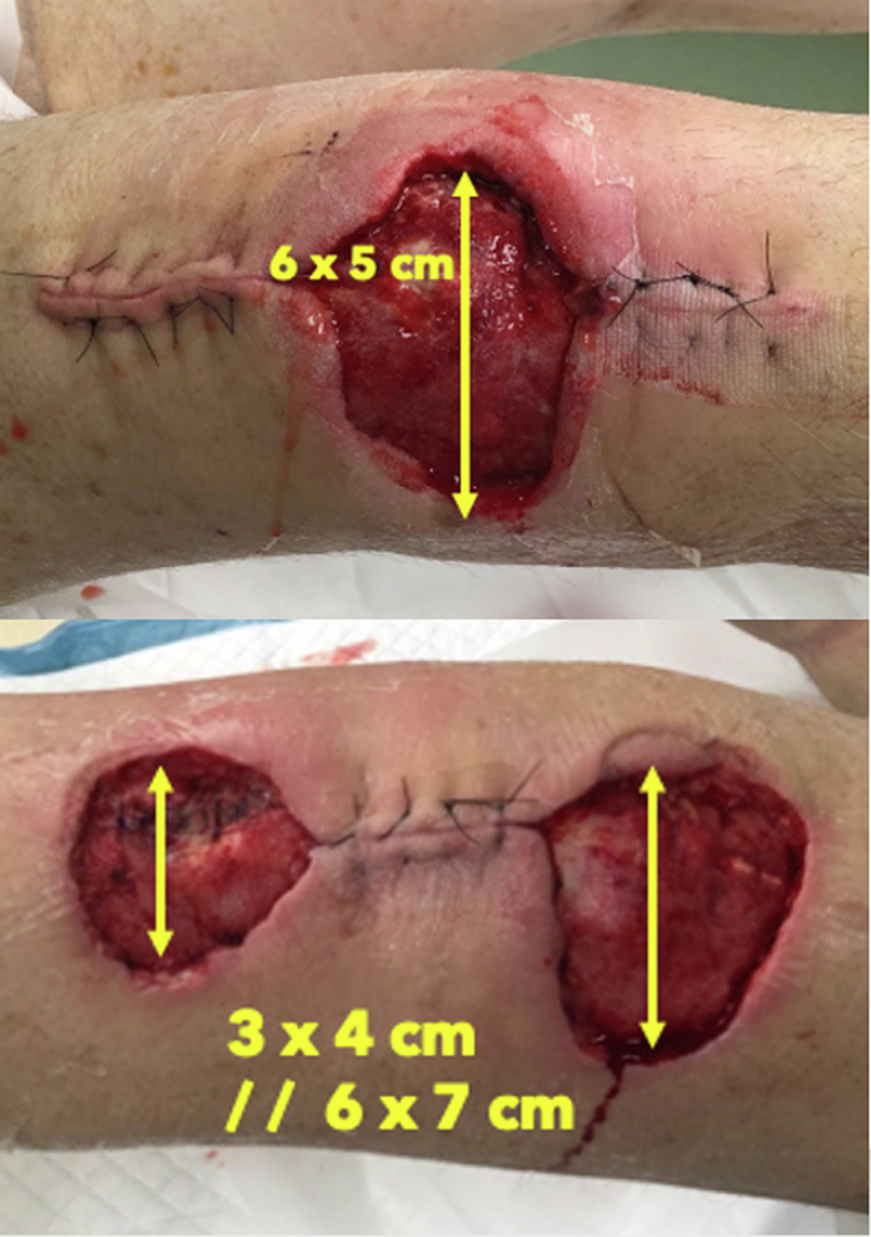
Bilateral knee wounds after operative debridement with working diagnosis of necrotizing soft-tissue infection.

Both knee aspirates were deemed negative for periprosthetic joint infection at this point. Alpha-defensin testing was also performed as a confirmatory test and was also found to be negative in both knees. However, based on wound appearance and the patient’s clinical deterioration, he was brought to the operating room for irrigation and debridement of both knees with a diagnosis of a necrotizing soft-tissue infection. This decision-making was supported by infectious disease consultation who agreed with the provisional diagnosis of necrotizing soft-tissue infection. Intraoperatively the patient had 3 separate areas of tissue necrosis which were debrided. The tissue was friable and avascular, requiring worrisome amounts of full-thickness skin removal to get to normal bleeding tissue. The right knee had a central skin paddle of 6 cm × 5 cm (30 cm^2^) removed, the left 3 cm × 4 cm (12 cm^2^) over the patellar tendon insertion, and 6 cm × 7 cm (42 cm^2^) over the distal quadriceps. There was no true purulence, and 5 separate specimens were taken for pathologic and microbiologic analysis (Fig. 5). Nine liters of normal saline was applied through pulse lavage during the irrigation of each knee, with arthrotomy closure using 1 PDS and dermal closure with 2-0 PDS. Negative pressure dressings were placed on the skin defects described previously (Kinetic Concepts, San Antonio, TX). Knee immobilizers were placed until further notice with the patient weight-bearing as tolerated for transfers and mobilization. After debridement and bilateral liner exchange, the patient continued to have elevated fever and WBC counts. All cultures remained negative from preoperative aspiration and intraoperative tissue cultures. Antibiotics were continued, and an antifungal agent was added.

**Figure 6:**
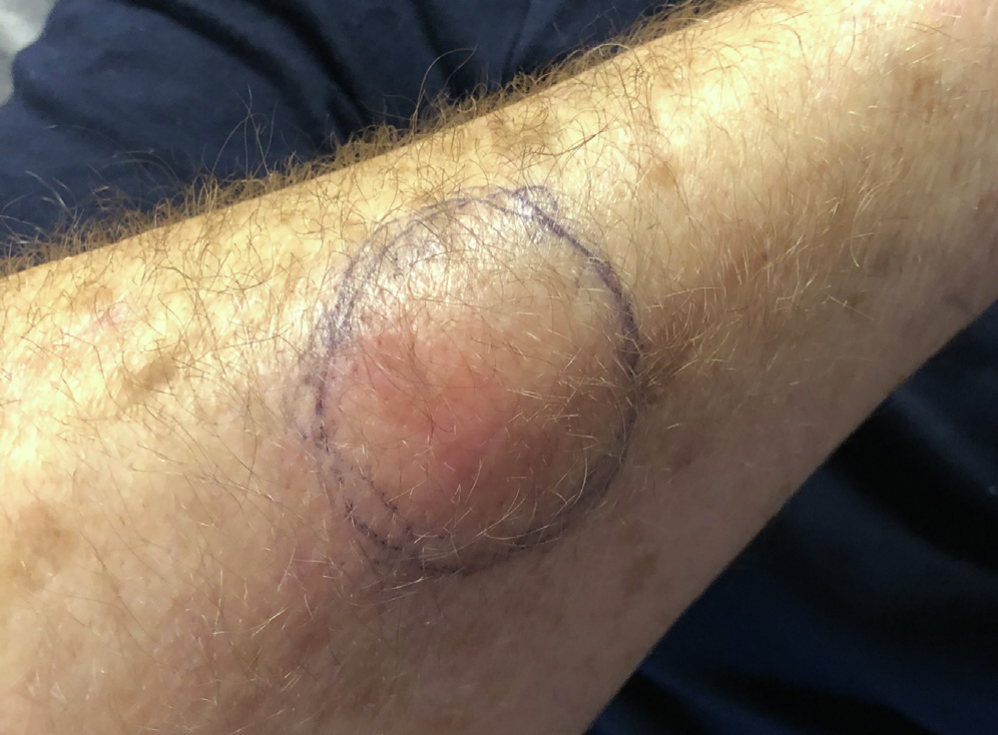
Site of pathergy identified at site of intravenous line placement.

**Figure 7:**
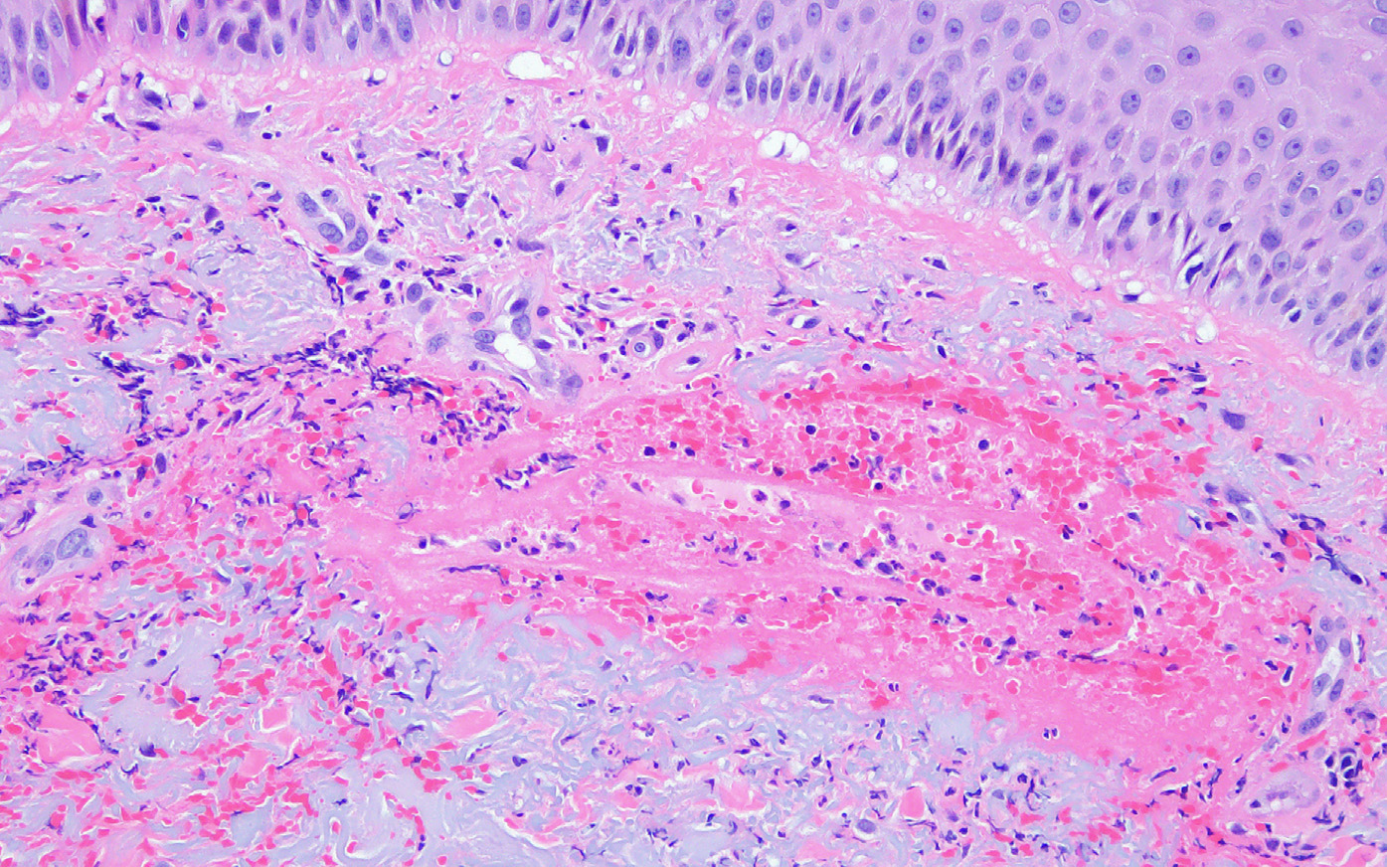
Microscopic analysis of wound edge showing dermal hemorrhage with superficial neutrophils and mixed dermal inflammation without evidence of vasculitis.

**Figure 8:**
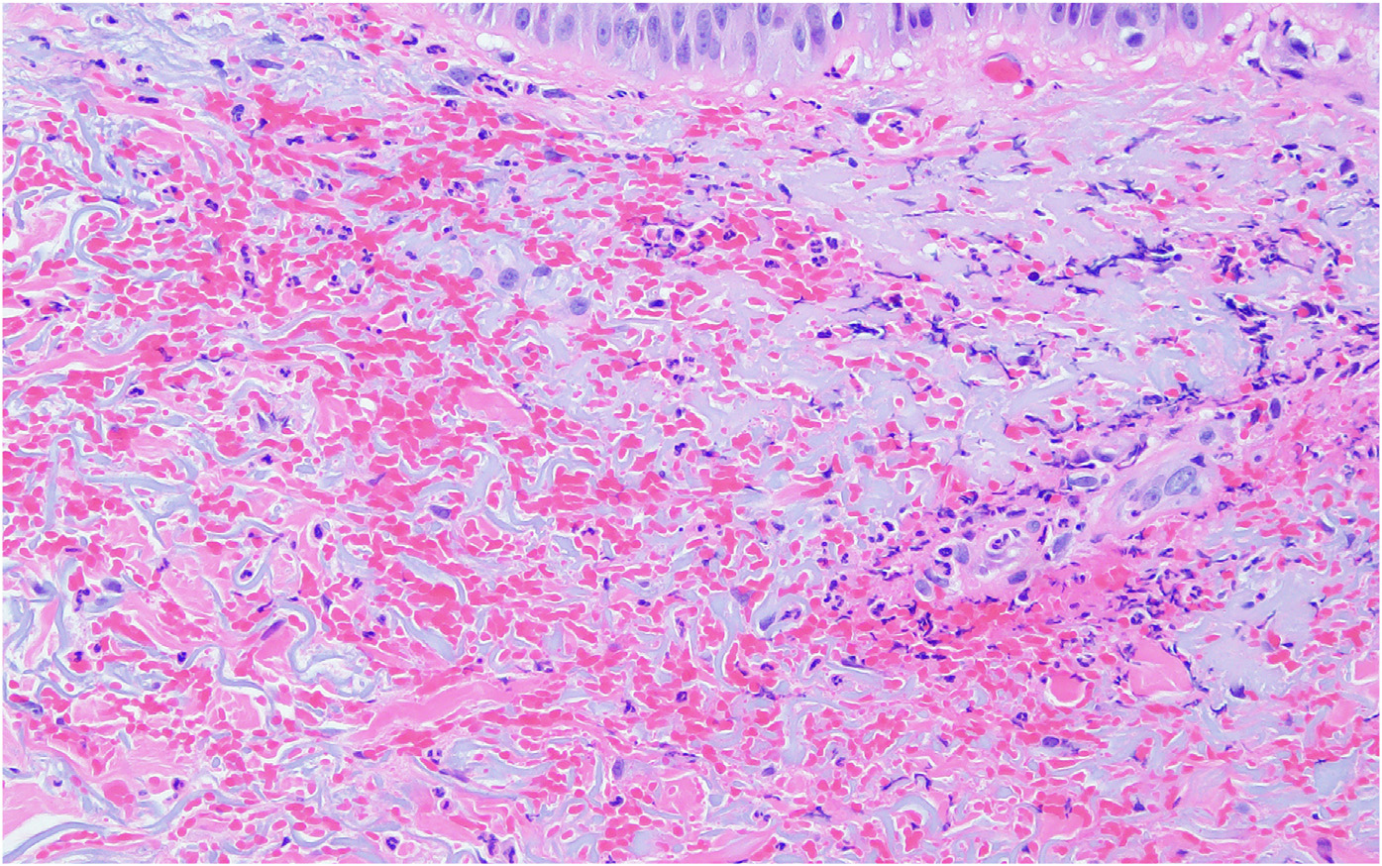
Microscopic analysis of wound edge showing dermal hemorrhage with superficial neutrophils and mixed dermal inflammation without evidence of vasculitis.

At this time, rheumatology consultation was obtained because of concern for an alternate diagnosis. Given the working diagnosis of necrotizing fasciitis, and as wound edges continued to show erythema and necrosis, the decision was made for a repeat trip to the operating room. Repeat debridement was superficial only as deep fascial layers were spared without adjacent purulence, cultures continued to show no growth, and pathologic analysis demonstrated only necrotic skin and PMNs. At this time, the patient agreed to be transferred to a tertiary academic care center with consultation from dermatology and plastic surgery. At this point, our team considered a wider range of noninfectious etiologies of wound breakdown, to include neutrophilic dermatosis. Dermatology had noticed an area of redness over a prior IV site (Fig. 6) and obtained forearm biopsy demonstrating “dermal hemorrhage with superficial neutrophils and mixed dermal inflammation without evidence of vasculitis” (Figure 7, Figure 8) with final diagnosis of PG based on these specimens in conjunction with aseptic knee aspiration and cultures. High-dose oral corticosteroid at 1 mg/kg/d was added with rapid improvement in his systemic picture as well as local wound appearance. He was maintained on oral corticosteroids and prophylactic oral antibiotics with negative pressure dressings applied.

**Figure 9:**
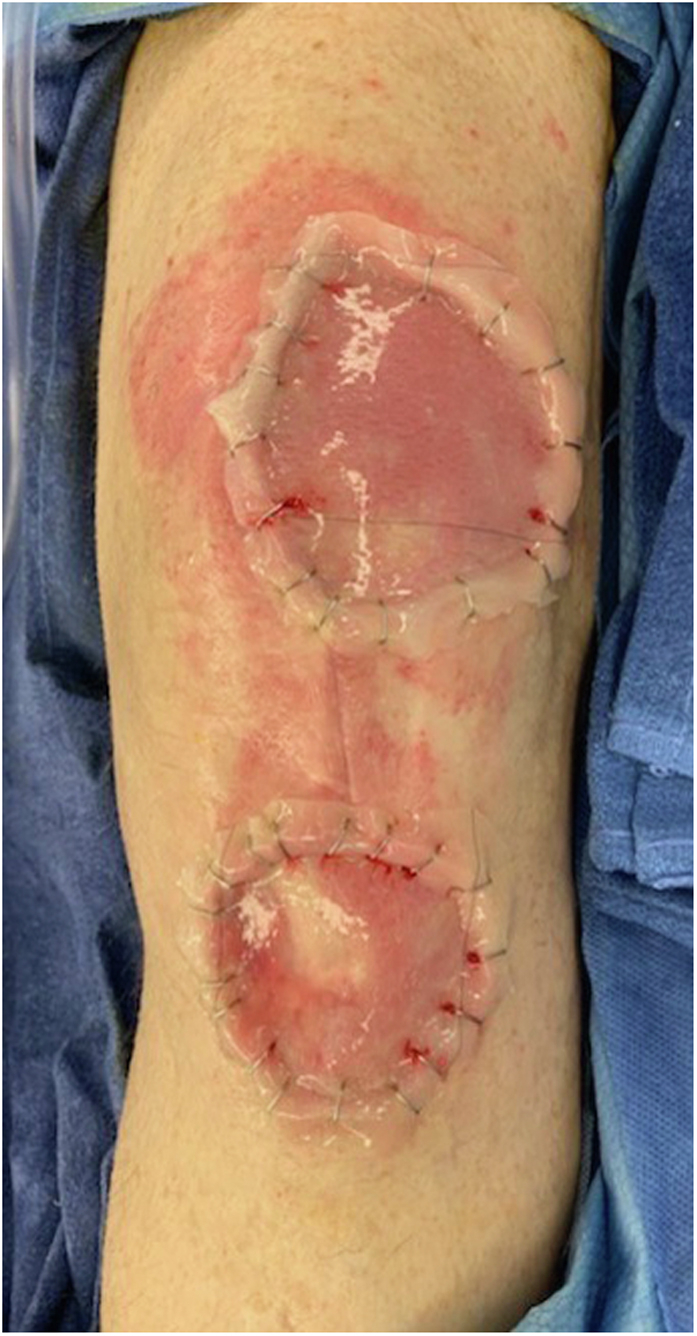
Left knee after diagnosis and control of pyoderma gangrenosum and placement of Integra skin substitute.

Plastic surgery consultation was obtained, and treatment options to include skin graft, Integra skin substitute (Integra LifeSciences, Princeton, NJ), rotational gastrocnemius flap(s), and free flap tissue transfer were considered. Eventual treatment with wound debridement and Integra Bilayer Matrix Wound Dressing grafting was performed followed by full-thickness skin grafting from the abdomen to bilateral knees (Fig. 9). The patient tolerated both procedures, and he continued on oral corticosteroids to suppress the neutrophilic dermatosis with continued monitoring by dermatology. Knee range of motion was limited to 90 degrees at this time to limit the strain on soft-tissue coverage.

**Figure 10:**
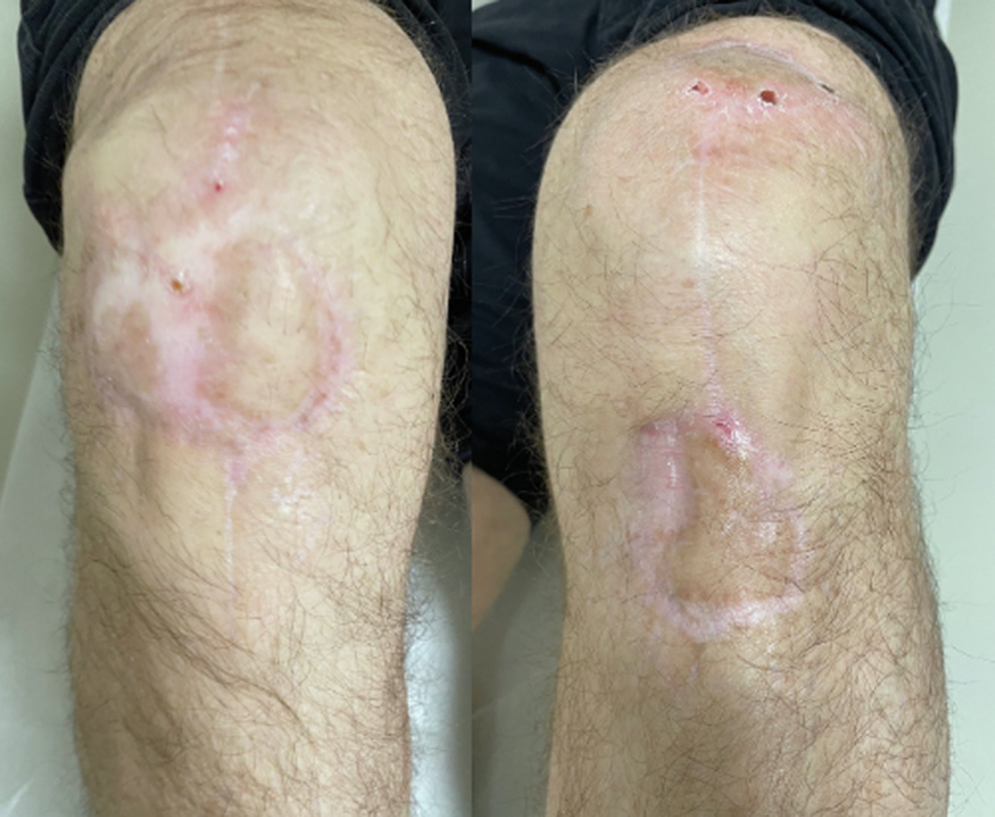
One-year clinical follow-up after with incorporation of Integra and split-thickness skin graft.

Fortunately, the Integra application with staged skin grafting was successful. At 6 weeks after soft-tissue coverage, he had gained excellent range of motion. At 8-month follow-up, the patient had been weaned off of prednisone therapy, and skin grafts had near-complete integration with a small area of incomplete healing over the patellar tendon of the left knee. Range of both knees was greater than 125 degrees, ambulation was unassisted, and the patient was cycling up to 50 miles per week with little discomfort. At 12-month follow-up, the patient had complete epithelialization of the infrapatellar wound (Fig. 10). From his perspective, the diagnosis and treatment of PG was quite stressful and delayed his recovery by at least 6 months. He felt fortunate in the degree of collaboration across specialists to care for the problem and now understands this autoimmune condition as part of his medical history.

## Discussion

PG is a noninfectious dermatologic condition whereby polymorphonuclear cells are upregulated and misguided, with the subsequent release of stimulating cytokines [12]. Through a process known as “pathergy,” PG can be set off secondary to soft-tissue trauma, which can include surgical procedures. A review in the plastic surgery literature has identified 220 cases of postsurgical PG during the years 1946-2013 [13]. The average time of PG presentation is 7 days after surgery, with time to diagnosis of 14.7 days [9]. Of these identified cases, 27 (12%) were seen after orthopedic surgeries, with 7 after knee arthroplasty and 4 after hip arthroplasty (Table 1).

**Table 1:** Case report review of pyoderma gangrenosum after joint arthroplasty.

Title and authors	Age/Sex	Medical Hx	Procedure	Presentation/Diagnosis	Treatment	Outcome
Hill et al. (2011) [14]	63 y/Female	Sjogren’s disease	UKA	POD 6/POD 19	NPT, HBO, immunosuppression, muscle flap.	Functional, chronic low-dose prednisone.
Nakajima et al. (2011) [15]	80 y/Female	Type II diabetes	TKA	POD 5/POD 17	Immunosuppression, muscle flap	Functional, minimal sequela
Wadia et al. (2006) [16]	80 y/Female	-	b/l TKA	POD 3/POD 8	Immunosuppression –healed on its own	Functional, minimal sequela
Verme (2009) [17]	72 y/Female	-	TKA	POD 3/POD 3	Immunosuppression, colloidal dressings	Functional, minimal sequela
Attar et al. (2010) [18]	80 y/Female	-	b/l TKA	POD 7/POD 7	Immunosuppression, wound Vac therapy	Unknown functional outcome
Yik et al. (2015) [19]	56 y/Male	-	TKA	POD 6/POD 15	Immunosuppression, medial gastrocnemius muscle flap coverage	No recurrence, unknown function outcome
Jain et al. (2005) [20]	63 y/Male	HTN	TKA	POD 6/POD 16	Split thickness skin graft before dx. Immunosuppression after diagnosis.	Improvement in lesions over time. Functional outcome not noted.

UKA, unicompartmental knee arthroplasty; POD, post operative day, NPT, negative pressure therapy; HBO, hyperbaric oxygen, TKA, total knee arthroplasty, HTN, hypertension.

This condition is problematic in regard to diagnosis, whereby it is often considered an infection or even necrotizing fasciitis at presentation, with 73% of cases in a large case series undergoing debridement for this reason. Subtle differences from infectious etiology can include a link with autoimmune conditions in the patient history (34% of patients), slower progression, and a characteristic violaceous wound border [9]. Most patients are diagnosed by exclusion after cultures return negative, and there is no response to antibiotic and surgical treatment. Dermatologic consultation and histologic evaluation are critical in confirming the diagnosis as well as response to corticosteroid therapies.

As suspicion mounts for PG in the postsurgical patient, corticosteroids should be considered as well as other immunosuppressive modalities. In this case, we used oral prednisone at 1 mg/kg/day and saw rapid improvement in both systemic symptoms and local wound condition. This dosing regimen was continued through the treatment of his local wounds and then gradually tapered over a course of 8 months. There are only case reports supporting treatments for soft-tissue coverage of postsurgical PG wounds in knee arthroplasty patients. Successful treatments have included local wound care, rotational muscle flaps, adjunctive hyperbaric oxygen, and free tissue transfer [13,21]. This case shows successful treatment with dermal substitute and full-thickness skin graft. At 1-year follow-up, this has proved successful in regard to soft-tissue coverage with limited harvest site morbidity compared with muscular transfer.

This case was challenged by the election of bilateral compared with unilateral total knee replacement, as well early aspiration pneumonitis which had the patient treated with empiric antibiotic therapy. As such, our clinical intuition was anchored on infectious etiologies when wound healing issues were identified about his knees. While these unexpected events made recovery from bilateral knee surgery more difficult, but both surgeon and patient agree he was an appropriate candidate for bilateral total knee replacement. A discussion of decision-making and risks of bilateral total knee replacement is beyond the scope of this report but does provide another data point of caution for those choosing bilateral total knee replacement. Rare entities such as this can become more challenging to recover from in the setting of bilateral surgery.

The decision of soft-tissue coverage method in this case considered his acute medical situation and comorbidities, morbidity associated with potential tissue transfers, and patient risk tolerance for these treatment pathways. The tension between the plastic surgeon and orthopedic surgeon included the prioritization of durable tissue coverage to protect the implant from infection vs the survivorship of tissue flaps in the setting of active PG. As such, through a collaborative decision and close clinical follow-up, our clinical team opted for dermal substitute coverage and split-thickness grafting. Tissue transfer was reserved as a second-line treatment should the initial treatment fail. Reserving substantial tissue transfer as a backup enabled control of the neutrophilic dermatosis and the avoidance of harvest site morbidity. It also allowed the patient to be treated for concomitant pulmonary emboli with necessary anticoagulants that increased the risk of flap hematoma. The left knee, which had >50 cm^2^ of tissue loss, likely would have required a free tissue transfer of latissimus dorsi, rectus abdominis, or other muscle of similar size. The right knee would have required a fully mobilized rotational medial gastrocnemius flap. Of note, one of the senior authors is a consultant for Integra Life Sciences, the maker of the skin substitute used in this case report.

Communication across multiple medical specialties and facilities was key in this case. Unfortunately, no members of the initial treatment had seen a case of active PG in their practice. Despite consultation with infectious disease and rheumatology teams in collaboration with pathology, our team did not hone in on the diagnosis of PG until more than 20 days after the operation. The diagnosis was obviously made after the initial debridement, which was in hindsight a radical treatment. Therein lies the challenge of neutrophilic dermatosis. As a pathologic entity that mimics infectious etiologies, there are arguably unnecessary trips to the operating room and patient morbidity. Guidance to surgeons with confusion in diagnosis of a poorly healing wound should consider consultation from multiple specialists—to include infectious disease, rheumatology, plastic surgery, and/or dermatology. Fortunately, teleconsultation services are more available now than in the past which can help with access to these specialties. Ideally our team would have identified this case as a neutrophilic dermatosis upon readmission to the hospital based on wound appearance with a violaceous border and the presence of high fevers. Earlier treatment with corticosteroid therapies as an alternative to, or in conjunction with, antibiotic therapies may have helped avoid the soft-tissue debridement which necessitated soft-tissue coverage.

In conclusion, postsurgical PG is a challenging problem in regard to diagnostic confirmation and because of the limited evidence behind definitive treatment. As PG mimics necrotizing fasciitis and other infectious etiologies, there is a strong recommendation for medical consultation from a dermatologic and/or rheumatology specialist for even a low suspicion. This may help avoid damaging tissue debridement and speed the time to treatment with high-dose corticosteroids, the latter of course counter to usual treatments for infection. Definitive treatment in regard to soft-tissue coverage should be assisted by a plastic surgeon with a consideration for use of local wound treatments and dermal substitute before advancing up the reconstructive ladder to rotational or free muscle flaps [13], [22], [23], [24].

## Conflicts of interest

Author Gordon Lee MD, senior author is a consultant for Integra Life Sciences. The authors declare that they have no known competing financial interests or personal relationships that could have appeared to influence the work reported in this article.

## Appendix A Supplementary data

Conflict of Interest Statement for Graw

Conflict of Interest Statement for Merrit

Conflict of Interest Statement for Lee

Conflict of Interest Statement for Loomis

Conflict of Interest Statement for Aleshin

Conflict of Interest Statement for Graw
