# Supplementary material for: Pyoderma Gangrenosum After Bilateral Total Knee Arthroplasty
Source: Arthroplast Today. 2021 Aug 26;11:73–9. doi: 10.1016/j.artd.2021.07.003 (PMC8397920; doi:10.1016/j.artd.2021.07.003)
Supplement: Conflict of Interest Statement for Lee [file mmc3.pdf]

CONFLICT OF INTEREST STATEMENT

American Association of Hip and Knee Surgeons

(Adopted from the American Academy of Orthopaedic Surgeons disclosure statement)

The following form **must be filled out completely and submitted by each author (example, 6 authors, 6 forms). All items require a response. If there is no relevant disclosure for a given item, enter "*None*."**

Manuscript Title: **Pyoderma Gangrenosum Following Bilateral Total Knee Arthroplasty**

1. Royalties from a company or supplier (The following conflicts were disclosed)

None

2. Speakers bureau/paid presentations for a company or supplier (The following conflicts were disclosed)

NONE

3A. Paid employee for a company or supplier (The following conflicts were disclosed)

None

3B. Paid consultant for a company or supplier (The following conflicts were disclosed)

Integra, Inc – Scientific Consultant

3C. Unpaid consultants for a company or supplier (The following conflicts were disclosed)

None

4. Stock or stock options in a company or supplier (The following conflicts were disclosed)

None

5. Research support from a company or supplier as a Principal Investigator (The following conflicts were disclosed)

None

6. Other financial or material support from a company or supplier (The following conflicts were disclosed)

None

7. Royalties, financial or material support from publishers (The following conflicts were disclosed)

Wolters Kluwer – Book royalties

8. Medical/Orthopaedic publications editorial/governing board (The following conflicts were disclosed)

None

9. Board member/committee appointments for a society (The following conflicts were disclosed)

Member At Large, American Society of Plastic Surgeons
Treasurer, California Society of Plastic Surgeons
Visiting Professor Committee Chair, ASPS
Leadership Development Committee, Co-Chair, ASPS
Program Committee, ASPS and CSPS

Each author must sign AND print or type his/her name, date and submit a separate form

In addition, one BLINDED Conflict of Interest form (no author names used) should be submitted per manuscript with all author disclosures.

Gordon K. Lee, MD FACS		4-27-21
Author Name (Print or Type)	Author Signature	Date
